# pH-Responsive Nanoparticles for Delivery of Paclitaxel to the Injury Site for Inhibiting Vascular Restenosis

**DOI:** 10.3390/pharmaceutics14030535

**Published:** 2022-02-27

**Authors:** Huiru Zhu, Li Kong, Xu Zhu, Tingting Ran, Xiaojuan Ji

**Affiliations:** 1 Department of Ultrasound Imaging, Children’s Hospital of Chongqing Medical University, Chongqing 400014, China; zhuhuiru@stu.cqmu.edu.cn (H.Z.); kongli123@stu.cqmu.edu.cn (L.K.); zhuxu@hospital.cqmu.edu.cn (X.Z.); ranting6@hospital.cqmu.edu.cn (T.R.); 2 National Clinical Research Center for Child Health and Disorders, Ministry of Education Key Laboratory of Child Development and Disorders, Chongqing Key Laboratory of Pediatrics, Chongqing 400014, China; 3 State Key Laboratory of Ultrasound in Medicine and Engineering, College of Biomedical Engineering, Chongqing Medical University, Chongqing 400016, China

**Keywords:** vascular restenosis, pH-responsive nanoparticles, sodium bicarbonate, paclitaxel, drug delivery

## Abstract

A high incidence of restenosis has been reported at the site of inflammation following angioplasty and stent implantation. The anti-proliferative drug paclitaxel (PTX) could help to reduce inflammation and restenosis; however, it has poor water solubility and serious adverse side effects at high doses. Given the presence of metabolic acidosis at the site of inflammation, we hypothesized that nanoparticles that are responsive to low pH could precisely release the loaded drug at the target site. We successfully constructed pH-responsive poly(D, L-lactic-co-glycolic acid) (PLGA) nanoparticles loaded with PTX and NaHCO_3_ as a pH-sensitive therapeutic agent (PTX-NaHCO_3_-PLGA NPs). The NPs exhibited remarkable pH sensitivity and a good safety profile both in vitro in rat vascular smooth muscle cells and in vivo in Sprague Dawley rats after tail vein injection. In the rat model, the PTX-NaHCO_3_-PLGA NPs treatment group showed suppressed intimal proliferation following balloon-induced carotid artery injury compared with that of the saline-treated control. Overall, these results demonstrate that our newly developed pH-responsive nanodrug delivery platform has the potential to effectively inhibit restenosis.

## 1. Introduction

The incidence of vascular stenotic diseases has been increasing. Angioplasty and stent implantation are the main clinical modalities for treating stenotic lesions; however, the high incidence of postoperative restenosis seriously affects the long-term prognosis of patients [1,2]. The process of balloon implantation into the vessel may disrupt endothelial cells, causing a local inflammatory response, with consequent recruitment of various inflammatory cells to produce inflammatory factors, triggering the proliferation and migration of vascular smooth muscle cells (VSMCs), and ultimately leading to intimal thickening. Paclitaxel (PTX) is an antiproliferative drug that can be used to prevent or treat restenosis [3,4,5,6,7]. Drug-eluting stents and drug-coated balloons can also effectively prevent vascular restenosis in clinical practice. However, long-term oral antiplatelet drugs are required after drug-eluting stents are implanted, otherwise the risk of thrombosis is increased [4]. In early 2022, Wang et al. reported a study of PTX-loaded coated stents for benign cicatricial airway stenosis, suggesting not only that PTX can be used to prevent restenosis but that it is equally effective for the airway. However, the study also illustrated that invasive stenting approaches can pose some problems, such as granulation tissue proliferation, which points to the need for noninvasive intervention modalities [5]. Moreover, systemic toxicity related to drug-coated balloons should also be noted [6]. However, due to disadvantages such as poor water solubility and toxic side effects that increase in a dose-dependent manner, the bioavailability and therapeutic effects of PTX are not satisfactory, as they lead to a series of adverse reactions [8]. Human VSMCs are highly sensitive to PTX, making it a suitable drug for inhibiting the proliferation of VSMCs [3]. However, restenosis has also been reported after implantation even with the use of drug-eluting stents [9,10]. Therefore, there is an urgent need to propose a new method of drug delivery to overcome these limitations.

Nanomedicine has been developing rapidly. As early as 2001, there were reports of PLGA wrapping Taxol to prevent restenosis [11]. However, it has not been studied in vivo. Juliana et al. designed targeted nanoparticles using natural slow-release drugs [12]. The pH response method adopted in this paper accelerates drug release. Poly (d, l-lactic-co-glycolic acid) (PLGA) can effectively solve the disadvantages of poor water solubility of PTX, and release the drug at a fixed point to reduce its toxic effect. PLGA [13,14] is a polymeric material with good bioavailability, which is synthesized from poly (lactic acid) and glycolic acid (i.e., ethanoic acid) in different ratios. PLGA polymers can be prepared in almost any size and morphology as well as modified into hydrophilic and hydrophobic molecules of almost any size; therefore, they are widely used for drug delivery. Polymeric materials with good biocompatibility have been used to construct nanoparticles for the specific targeted delivery of drugs or as contrast agents for detecting tumors [15,16], as well as cardiovascular [17,18], neurological [19,20], and other diseases. Based on the low immunogenicity of polyethylene glycol (PEG) [21], PLGA-PEG, a block co-polymer, is more suitable for vivo experiments than PLGA alone [22]. Additionally, the fastest drug release can be achieved at a lactic-acid-to-glycolic-acid ratio of 50:50 [23].

The physiological extracellular pH is 7.4, whereas the pH at the site of vascular inflammation is reduced, making the microenvironment slightly acidic. Metabolic acidosis at the site of inflammation also contributes to the formation of an acidic microenvironment [24]. Sodium bicarbonate (NaHCO_3_) reacts with protons in an acidic environment to produce salt and carbonic acid, which in turn can be broken down into CO_2_ and water [25].

**Scheme 1:**
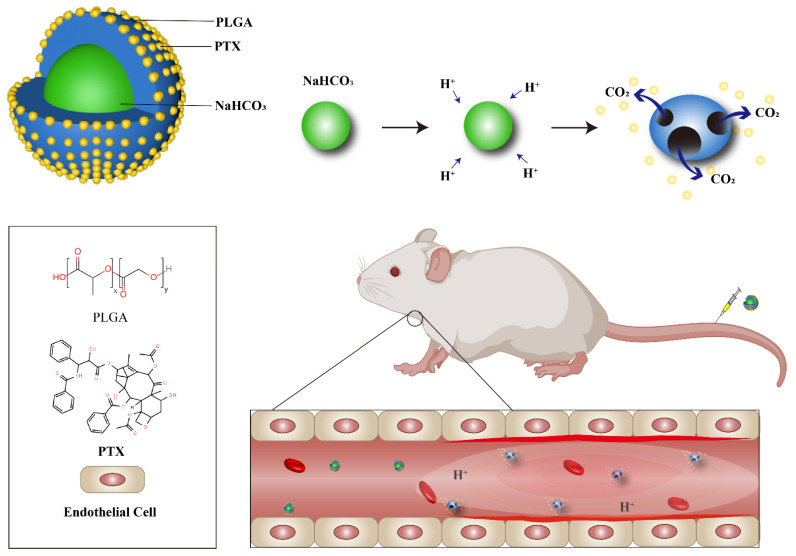
Design of pH-responsive nanoparticles for the treatment of vascular restenosis.

Based on these chemical properties of PLGA and NaHCO_3_ and to enhance the drug targeting of PTX, we constructed a pH-responsive nanomaterial comprising PLGA nanoparticles loaded with PTX and NaHCO_3_ as a pH-sensitive therapeutic agent (PTX-NaHCO_3_-PLGA NPs) to accelerate drug release in an acidic microenvironment. Specifically, the pH-sensitive component NaHCO_3_ was encapsulated in PLGA, which could disintegrate the PTX-NaHCO_3_-PLGA NPs and could release the drug only when it reached the acidic microenvironment (Scheme 1) [25,26].

In this study, we synthesized and characterized PTX-NAHCO_3_-PLGA NPs, and performed in vitro and in vivo safety tests using rat VMSCs and a rat model of carotid artery injury with a balloon, respectively. Furthermore, we evaluated the therapeutic efficacy and biocompatibility of the PTX-NAHCO_3_-PLGA NPs in the animal model. These results highlight the potential of pH-responsive nanomaterials as a good drug delivery platform to target the injury site and to release drugs to treat revascularization.

## 2. Materials and Methods

### 2.1. Materials

PEG-PLGA-COOH (50:50, MW: 15,000) was purchased from Daigang BIO Engineer Ltd., Co. (Shan Dong, China). Poly (vinyl alcohol) (PVA) was purchased from Sigma-Aldrich Chemical Co., Ltd., (St. Louis, MO, USA); trichloromethane (CHCl3) was purchased form Chuandong Chemical Industry Group Co., Ltd., (Chongqing, China); NaHCO_3_ was purchased from Aladdin Biochemical Technology Co., Ltd., (Shanghai, China); PTX was purchased from Aladdin Biochemical Technology Co., Ltd., (Shanghai, China); BCECF-AM was purchased form Beyotime Biotechnology (Shanghai, China). The Cell Counting Kit (CCK-8) was purchased from Dojindo Laboratories (Kumamoto, Japan). A 2F Fogarty embolectomy catheter was purchased from Baxter Edwards Healthcare Corp. (Irvine, CA, USA).

### 2.2. Preparation of Nanoparticles

NaHCO_3_-PLGA NPs were prepared using the ultrasound double emulsification method (water/oil/water, W/O/W). First, 40 mg PEG-PLGA-COOH (50:50, MW: 15,000) and 4 mg PTX were dissolved in 2 mL trichloromethane (CHCl_3_). Once completely dissolved, 200 mL of 0.25% NaHCO_3_ aqueous solution was added. The mixture was processed using an ultrasonic cell crusher (VCX150, Sonics & Materials, Inc., Newtown, CT, USA) at 105 W for 1 min 30 s to obtain the W/O emulsion. Next, 4 mL of 4% poly(vinyl alcohol) (PVA) aqueous solution was added [27] and the mixture was emulsified using an ultrasonic cell crusher at 105 W for 2 min 30 s to obtain W/O/W emulsion. Subsequently, 10 mL of a 2% isopropyl alcohol solution was added and the mixture was stirred for 6 h to volatilize the CHCl_3_. Finally, the PTX-NaHCO_3_-PLGA NPs were collected by centrifugation several times (10,000 rpm, 8 min).

### 2.3. Characterization of PTX-NaHCO_3_-PLGA NPs

The nanoparticles were diluted with neutral PBS and the size distribution and zeta potential of PTX-NaHCO_3_-PLGA NPs were measured using a Nano ZS90 Zetasizer (Malvern Panalytical, Ltd., Malvern, UK). Scanning electron microscopy (SEM, S-3400N, Hitachi, Ltd., Tokyo, Japan) and transmission electron microscopy (TEM; HT7500, Hitachi, Ltd., Tokyo, Japan) were employed to observe the structure of the PTX-NaHCO_3_-PLGA NPs. The value of the pH of the PTX-NaHCO_3_-PLGA NPs dissolved in neutral PBS was measured using a pH meter (SevenCompact, Mettler Toledo, Columbus, OH, USA). The molecular structure and chemical bonding characteristics of freeze-fried PTX-NaHCO_3_-PLGA NPs were analyzed using Fourier transform infrared spectroscopy (FTIR) (Nicolet™ iS50, Thermo Fisher Scientific, Ltd., Waltham, MA, USA).

### 2.4. Drug Loading Content, Encapsulation Efficiency, and Release Determination

Using the method reported by Gui et al. [28], the standard curve of PTX was obtained using an ultraviolet (UV) spectrophotometer (Shimadzu UV 2600, Shimadzu Corporation, Kyoto, Japan). The nanoparticles were destroyed with an organic solvent (dimethyl sulfoxide: methanol = 1:1), and the concentration of PTX was determined and analyzed. According to the method of Mahdi et al. [29], the encapsulation and drug loading rates of PTX were calculated as follows:

$$\mathrm{EE}\;\left(\%\right)=\frac{\mathrm{amount}\;\mathrm{of}\;\mathrm{drug}\;\mathrm{applied}\;\mathrm{for}\;\mathrm{nanoparticle}\;\mathrm{preparation}\;\left(\mathrm{mg}\right)\;-\;\mathrm{amount}\;\mathrm{of}\;\mathrm{drug}\;\mathrm{in}\;\mathrm{the}\;\mathrm{supernatant}\;(\mathrm{mg})}{\mathrm{amount}\;\mathrm{of}\;\mathrm{drug}\;\mathrm{applied}\;\mathrm{for}\;\mathrm{nanoparticle}\;\mathrm{preparation}\;(\mathrm{mg})}\times 100\%$$

$$\mathrm{DL}\;\left(\%\right)=\frac{\mathrm{amount}\;\mathrm{of}\;\mathrm{drug}\;\mathrm{applied}\;\mathrm{for}\;\mathrm{nanoparticle}\;\mathrm{preparation}\;\left(\mathrm{mg}\right)\;-\;\mathrm{amount}\;\mathrm{of}\;\mathrm{drug}\;\mathrm{in}\;\mathrm{the}\;\mathrm{supernatant}\;(\mathrm{mg})}{\mathrm{amount}\;\mathrm{of}\;\mathrm{dried}\;\mathrm{nanoparticle}\;(\mathrm{mg})}\times 100\%$$

To characterize PTX release, PTX-NaHCO_3_-PLGA NPs (containing 5 mg PLGA) were dissolved in 1 mL neutral PBS, and then were dispersed in phosphate-buffered saline (PBS; 50 mL) at pH 5, pH 6, or pH 7.4. The suspended materials were added into a dialysis bag (MWCO = 8000–14,000 Da) and stirred at 200 rpm at 37 °C. At different time points, the outside PBS (1 mL) was replaced with fresh PBS and subjected to PTX detection using the UV spectrophotometer.

### 2.5. Cell Culture

Rat-derived VSMCs were purchased from iCell Bioscience Inc. (Shanghai, China). The cells were maintained at 37 °C with 5% CO_2_ in Dulbecco’s Modified Eagle Medium supplemented with 10% fetal bovine serum and 1% streptomycin/penicillin.

### 2.6. In Vitro Cytotoxicity Assay

To evaluate the safety of PTX-NaHCO_3_-PLGA NPs in vitro, we co-incubated different concentrations of NPs (0.1, 0.5, 1.0, 5.0, 10, and 15 mg/mL) with rat VSMCs at 37 °C overnight, and cell viability was assessed using the standard Cell Counting Kit (CCK-8; *n* = 6), according to the following equation:

Cell viability (%)=(dosing group−blank group) ÷ (nondosing group−blank group)×100%)

### 2.7. Animal Model and Treatment

Twenty-five male Sprague Dawley (SD) rats (270–320 g), obtained from the Laboratory Animals of Chongqing Medical University, were divided into a sham-operated group (normal), a saline-treated group (model), and groups treated with NaHCO_3_-PLGA NPs, free PTX, and PTX-NaHCO_3_-PLGA NPs, respectively (*n* = 5 per group). All animal care and use procedures were reviewed and approved by the Animal Ethics Committee of Chongqing Medical University.

After intraperitoneal anesthesia with 3% pentobarbital sodium (0.1 mL/100 g), the ventral part of the rat was fixed onto the animal operating table. A median cervical incision was made to separate the subcutaneous and muscular tissue layer by layer, and the left common carotid artery, external carotid artery, and internal carotid artery were fully exposed. A 2F Fogarty embolectomy catheter was inserted into the external carotid artery, and the common carotid artery was damaged. Finally, the tissue was sutured layer by layer. Penicillin was injected intramuscularly for 3 days after the operation to help prevent infection [30].

Treatments were administered immediately after model establishment, and on days 5 and 10 after establishment. PTX (1 mg/kg) was administered to the rats via tail vein injection [31].

### 2.8. Hemolysis Assay

For the hemolysis test, blood samples were collected from the rats; then, erythrocytes were obtained by 10 min centrifugation at 3500 rpm and 4 °C, and washed with sterile isotonic PBS three times. One hundred microliters of erythrocytes were incubated with PTX-NaHCO_3_-PLGA NPs containing different drug concentrations (0, 8, 16, 32, 64, 128, and 256 μg/mL) for 4 h at 37 °C. The cells were then centrifuged for 3 min at 3500 rpm and 4 °C, the supernatant was collected, and the absorbance at 541 nm was read by UV-visible spectrophotometry. Erythrocytes dispersed in PBS were used as positive controls and erythrocytes dispersed in deionized water served as a negative control. The hemolysis rate was calculated using the following formula:

$$\begin{array}{ll} \mathrm{Hemolysis}\;\mathrm{rate}\;\left(\%\right) & \\ & =\left(\mathrm{treatment}\;\mathrm{group}-\mathrm{negative}\;\mathrm{control}\;\mathrm{group}\right)÷(\mathrm{positive}\;\mathrm{control}\;\mathrm{group}\\ & -\mathrm{negative}\;\mathrm{control}\;\mathrm{group})\times 100\% \end{array}$$

### 2.9. Detection of pH at the Site of Carotid Endothelial Injury

Carotid arteries from normal rats and injured vessels from the model rats were harvested on days 4, 7, 10, and 14 after modeling and embedded in OCT compound. Frozen sections were incubated with 2′,7′-bis-(2-carboxyethyl)-5-(and-6)-carboxyfluorescein-acetoxymethyl solution (BCECF-AM) for 30 min, and washed three times with PBS, sealed, and photographed under a laser-scanning confocal microscope (STELLARIS 5, Leica, Germany).

### 2.10. Therapeutic Efficacy of PTX-NaHCO_3_-PLGA NPs

The rats were euthanized on day 14 after injury, and the injured vessels were fixed in 4% paraformaldehyde, paraffin-embedded, sectioned, and routinely stained with hematoxylin and eosin (H&E). The sections were photographed by optical microscopy (BX51 optical microscope, Olympus, Corp., Tokyo, Japan), and images were digitally analyzed by ImageJ software (National Institutes of Health, Bethesda, MD, USA). The intimal and medial areas were measured, and the intima hyperplasia index was calculated as the ratio of the intimal area to the medial area [32,33].

In addition, the neck tissue sections were stained with antibodies against alpha-smooth muscle actin (αSMA) and proliferating cell nuclear antigen (PCNA), photographed by light microscopy, and quantitatively analyzed using ImageJ.

### 2.11. Biocompatibility Evaluation of PTX-NaHCO_3_-PLGA NPs

Male SD rats (250–300 g) were divided into 3 groups (NP, PTX and control groups) (*n* = 3 per group) and injected with 1 mL PTX-NaHCO_3_-PLGA NPs (2 mg/kg of PTX), 1 ml free PTX (2 mg/kg of PTX), or 1 mL PBS via the tail vein, respectively. Blood samples were collected on the first day after injection (control group), and on days 3, 7, and 14 for blood biochemical assays. The major organs (heart, liver, spleen, lungs, and kidneys) were collected, and tissue sections were prepared and stained with H&E.

### 2.12. Statistical Analysis

All data are expressed as mean ± standard deviation. GraphPad Prism (version 9.0) was used for statistical analysis. Paired *t*-tests and one-way analysis of variance were conducted to analyze the data. Results with *p* < 0.05 were considered statistically significant.

## 3. Results and Discussion

### 3.1. Synthesis and Characterization of PTX-NaHCO_3_-PLGA NPs

PEG-coated NPs not only avoid plasma protein adsorption, but also minimize interactions with phagocytes and increase blood circulation time [34,35]. This new specific drug delivery method has greater advantages than traditional intravenous or oral delivery, such as the controlled release of drugs, better targeting ability, improved bioavailability of drugs, and reduced damage to normal tissues [36,37,38].

**Figure 1:**
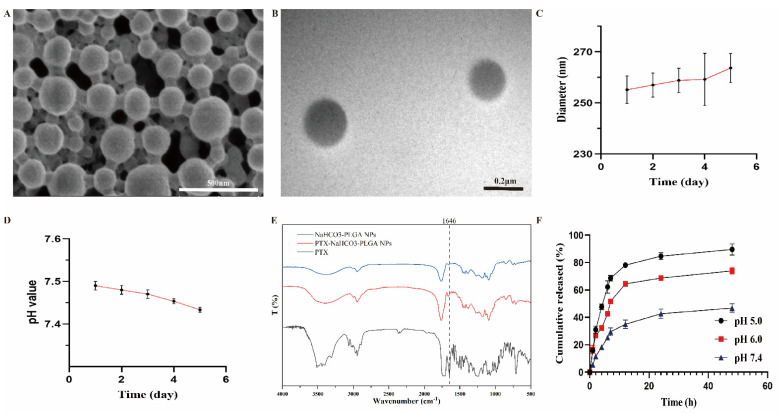
Characterization of PTX-NaHCO_3_-PLGA NPs. (**A**) SEM image and (**B**) TEM image of PTX-NaHCO_3_-PLGA NPs. (**C**) The size distribution and (**D**) pH value of PTX-NaHCO_3_-PLGA NPs in neutral PBS with prolonged time duration (*n* = 3). (**E**) Release profiles of PTX from PTX-NaHCO_3_-PLGA NPs at pH 7.4, 6.0, and 5.0, respectively. (**F**) FTIR of PTX, NaHCO_3_-PLGA NPs, and PTX-NaHCO_3_-PLGA NPs.

We successfully constructed PTX-NaHCO_3_-PLGA NPs using the double emulsion method. The nanoparticles had regular and spherical shapes under SEM and TEM observation (Figure 1A,B). The particle size of PTX-NaHCO_3_-PLGA NPs measured using a Malvern particle size meter was 255.2 ± 7.7 nm, along with a relatively narrow size distribution and an average zeta potential of –12.0 ± 2.4 mV, which means these nanoparticles have good physical stability [39]. The drug loading and encapsulation rates of PTX were 73.6 ± 3.4% and 4.4 ± 0.2%, respectively. Meanwhile, within 5 days, the mean particle size of PTX-NaHCO_3_-PLGA NPs did not appreciably change when dissolved in PBS (Figure 1C), revealing the excellent stability of the PTX-NaHCO_3_-PLGA NPs. Although the pH value of the nanoparticles decreased in 5 days, it did not change by more than 0.1 and remained weakly alkaline. Therefore, we believe that the -COOH group will not lead to acidic hydrolysis of PTX-NaHCO_3_-PLGA NPs (Figure 1D).

As shown in Figure 1E, the characteristic peaks of PTX at 1646 cm^−1^ were due to C=O stretching [40]. Relative to the PTX alone, the stretching vibration peak of the formed PTX-NaHCO_3_-PLGA NPs shifted to 1663 cm^−1^ at 1645 cm^−1^ for the amide group, indicating that there was partial interaction between PTX and PEG [41].

In neutral PBS buffer, the drug was released slowly, and the cumulative percentage of drug release was <50%. However, PTX release was significantly accelerated in a slightly acidic environment (pH 5.0, 6.0) compared with that observed in a neutral environment (pH 7.4), and more than half of the drug was released within 24 h (Figure 1F). This suggested that PTX-NaHCO_3_-PLGA NPs have good pH responsiveness and exhibit desirable pH-responsive drug release ability. Thus, we successfully constructed pH-responsive nanoparticles using materials such as NaHCO_3_ and PLGA.

In vitro drug release from pH-responsive drug-loaded nanoparticles mimics the conditions of in vivo vascular inflammatory diseases with a slightly acidic environment at the lesion site. Thus, the pH-responsive release behavior of the nanodrugs was consistent with our expectations and demonstrated the potential of PTX-NaHCO_3_-PLGA NPs for the prevention and treatment of vascular restenosis.

### 3.2. Biosafety of PTX-NaHCO_3_-PLGA NPs

**Figure 2:**
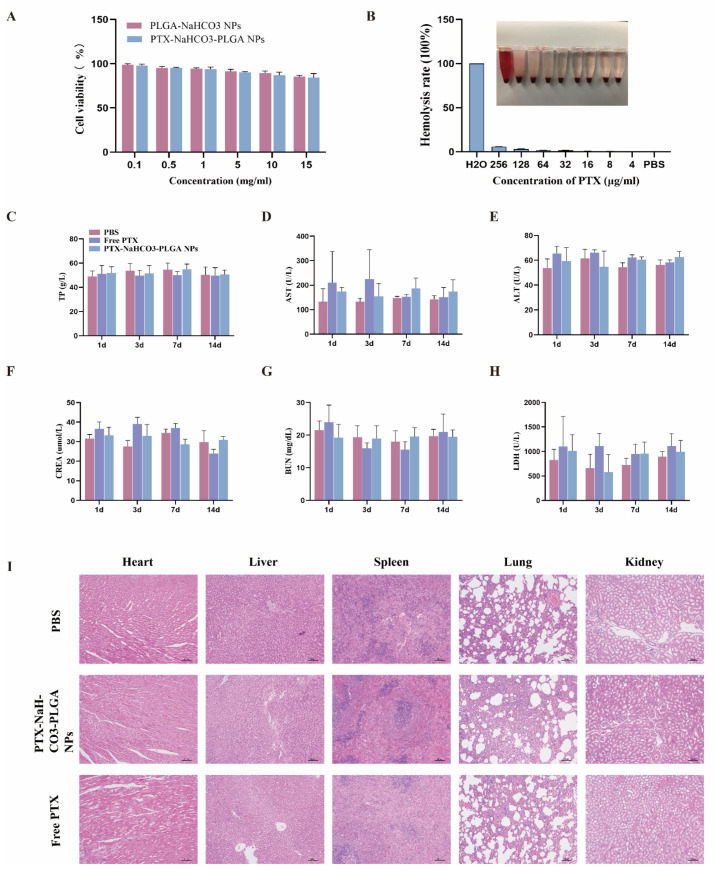
Biosafety evaluation of PTX-NaHCO_3_-PLGA NPs. (**A**) Cell viability of VSMCs with various concentrations of NPs. (**B**) Hemolysis test of PTX-NaHCO_3_-PLGA NPs. (**C**–**H**) Blood biochemical index results (ALT, AST, TP, CREA, BUN, and LDH). (**I**) H&E-stained sections of major organs in the PBS and PTX-NaHCO_3_-PLGA NPs groups. Scale bar: 100 μm. TP, total protein; AST, aspartate aminotransferase; ALT, alanine aminotransferase; CREA, creatinine; BUN, blood urea nitrogen; LDH, L-lactate dehydrogenase. Data are presented as mean ± SD (*n* = 3).

We first evaluated the cytotoxicity of different NPs in rat VSMCs. For PTX-NaHCO_3_-PLGA NPs and NaHCO_3_-PLGA NPs, cytotoxicity was detected at different drug concentration doses (Figure 2A). Even at the maximum concentration of 200 mg/mL, a high cell-survival rate was observed. In a microenvironment with pH 7.4, the PTX-NaHCO_3_-PLGA NPs were more stable and did not release too much PTX, leading to low cytotoxicity. This indicated the good biocompatibility and selectivity of the nanoparticles.

To evaluate the in vivo safety of PTX-NaHCO_3_-PLGA NPs, we performed hemolysis and blood biochemistry tests. The hemolysis rate reached 5.88 ± 0.02% when the PTX concentration was 256 μg/mL, and the PTX-NaHCO_3_-PLGA NPs showed good safety at all other concentrations (Figure 2B).

The blood biochemistry parameters in the PTX-NaHCO_3_-PLGA NPs and free PTX groups were not significantly different from those of the PBS group (Figure 2C–H). In addition, no significant damage was observed in major organs such as the heart, liver, spleen, lungs, and kidneys based on H&E staining (Figure 2I). Based on these results, we concluded that PTX-NaHCO_3_-PLGA NPs have a good biosafety profile.

### 3.3. pH Measurement at the Site of Vascular Injury

Restenosis is a repair response to damage in the vessel wall. Stent implantation causes strain and compression of the vessel, resulting in intimal damage, platelet aggregation activation, and the release of platelet-derived growth factor and inflammatory factors, thereby inducing VSMCs to undergo phenotypic modulation and change from a contractile to a synthetic phenotype, leading to their proliferation and migration. Stents are recognized as foreign bodies and activate the immune response by causing inflammatory cell aggregation, as well as the activation and release of inflammatory factors, ultimately leading to intimal thickening [42,43]. Many studies have shown that inflammation is central to intimal hyperplasia after arterial injury [44,45].

**Figure 3:**
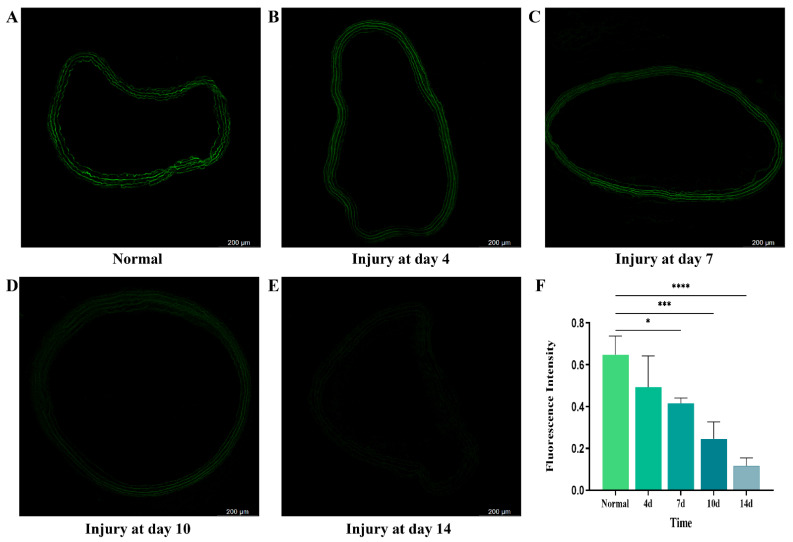
Fluorescence images and fluorescence analysis of vascular sections stained with the pH-sensitive fluorescent probe BCECF AM. (**A**–**E**) Fluorescence images at different time points after injury. (**F**) Quantitative analysis of fluorescence intensity of vascular sections. Data in (**F**) are presented as mean ± SD. * *p* < 0.05, *** *p* < 0.001, **** *p* < 0.0001.

Acute and chronic inflammatory tissues often exhibit metabolic acidosis due to the accumulation of lactic acid [46,47]. However, there are no definitive studies to confirm the presence of an acidic microenvironment in the arteries after balloon injury. We used the pH-sensitive fluorescent probe BCECF-AM to stain the carotid arteries before and after injury to assess the pH changes [48]. As shown in Figure 3, normal arteries showed green fluorescence, and its intensity gradually decreased with an increase in time, indicating that the pH of the injury site gradually decreased over time. On the 14th day after the injury, the intensity of green fluorescence in the intima decreased significantly, indicating that the pH of the injury site was significantly reduced, turning the microenvironment acidic; this may be related to the inflammatory response caused by balloon injury and the consequent production of various acidic metabolites.

### 3.4. Therapeutic Effects of PTX-NaHCO_3_-PLGA NPs

VSMCs have an almost undetectable proliferation index under normal physiological conditions. However, when the intima is injured, many inflammatory factors are released, and VSMCs undergo pathological changes, and abnormal migration, proliferation, and differentiation, ultimately transforming into proliferative VSMCs that participate in vascular reconstruction and cause restenosis [45]. Previous studies have found that PTX can effectively inhibit the migration and proliferation of VSMCs [31,49]. Therefore, PTX is a candidate drug for preventing restenosis.

**Figure 4:**
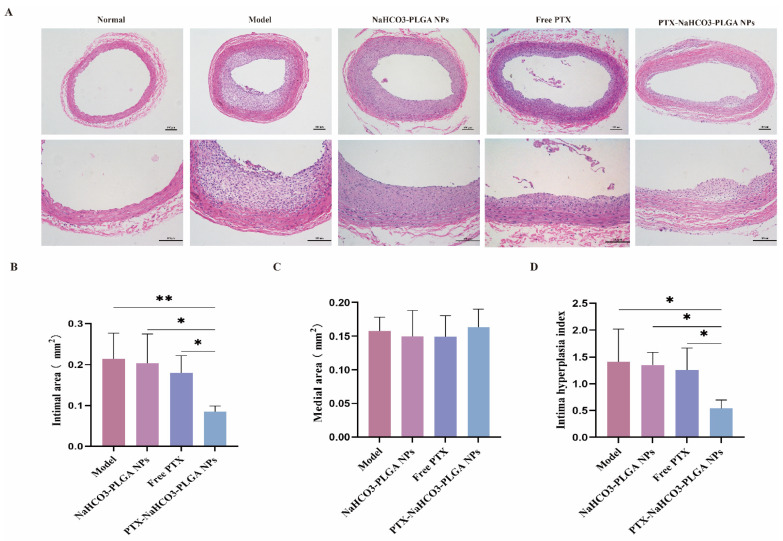
Evaluation of the efficacy of PTX-NaHCO_3_-PLGA NPs in the treatment of vascular restenosis. (**A**) H&E staining of blood vessel sections. Scale bar: 100 μm. (**B**–**D**) Quantitative analysis of the intimal area (**B**), medial area (**C**), and intimal hyperplasia index (**D**) of carotid arteries between four groups. Data in (**B**–**D**) are presented as mean ± SD (*n* = 5). * *p* < 0.05, ** *p* < 0.01.

We investigated the efficacy of PTX-NaHCO_3_-PLGA NPs in vivo. After balloon injury, the rats were administered saline or PTX-NaHCO_3_-PLGA NPs. On day 14 after treatment, H&E-stained images of the carotid arteries showed that the lumen of normal vessels was patent, with no intimal hyperplasia. In the model group treated with saline, the lumen was significantly narrowed and substantial endothelial hyperplasia was observed (Figure 4A). However, in the PTX-NaHCO_3_-PLGA NPs group, endothelial hyperplasia was markedly inhibited and the lumen was enlarged. Moreover, significantly weaker intimal hyperplasia assembly was observed in the PTX-NaHCO_3_-PLGA NP-treated group compared with that in the saline-treated group. As observed in the H&E sections, the area of the intima was reduced in the free PTX and NaHCO3-PLGA NPs treatment groups compared with the model group, but no statistical difference was observed between them, as shown by quantitative analysis. The toxic effects of PTX are known to increase with increasing dose [8]. When PTX was maintained at a safer dose, it had a limited treatment of revascularization, inferior to PTX-NaHCO_3_-PLGA NPs.

Moreover, quantitative analysis of H&E-stained sections revealed that the PTX-NaHCO_3_-PLGA NP treatment significantly inhibited endothelial proliferation and increased the lumen size. The intima area was significantly larger (*p* < 0.01; Figure 4B) in the PTX-NaHCO_3_-PLGA NP treatment group than that in the saline group, suggesting that PTX-NaHCO_3_-PLGA NPs effectively inhibited endothelial proliferation. However, the differences in the medial area between the two groups were not statistically significant (Figure 4C). In addition, the intima hyperplasia index, a measure of intimal proliferation, was significantly lower in the PTX-NaHCO_3_-PLGA NP-treated group than in the other three groups (Figure 4D).

**Figure 5:**
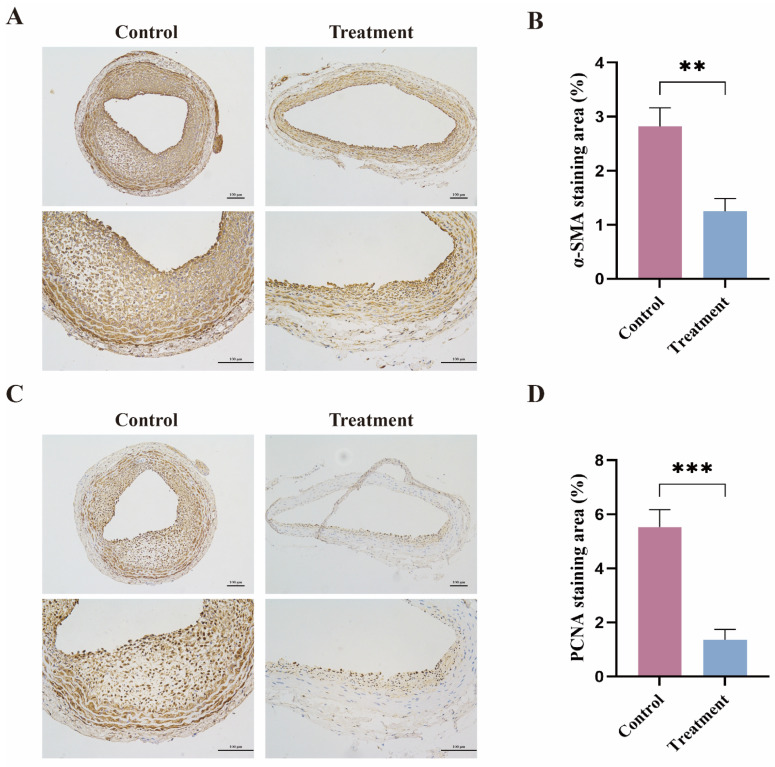
Immunohistochemical analysis of cross-sections of carotid arteries between two groups. (**A**) Microscopic images showing sections stained with antibodies to α-SMA. Scale bar: 100 μm. (**B**) Quantitative analysis of the percent of α-SMA staining area. (**C**) Microscopic images showing sections stained with antibodies to PCNA. Scale bar: 100 μm. (**D**) Quantitative analysis of the percent of PCNA staining area. Data in (**B**,**D**) are presented as mean ± SD (*n* = 5). ** *p* < 0.01, *** *p* < 0.001.

The anti-stenotic effect of PTX-NaHCO_3_-PLGA NPs was further evaluated by immunohistochemical staining. The positively stained area of α-SMA, as a typical marker of VSMCs, in the intima was significantly larger in the saline group than in the PTX-NaHCO_3_-PLGA NP-treated group (*p* < 0.05; Figure 5A,B), which partly confirmed that the proliferation and migration of VSMCs promoted the proliferation of the endothelium, whereas PTX effectively inhibited the proliferation and migration of VSMCs. Staining for PCNA, a cell proliferation marker, showed that proliferating cells were widely distributed in the endothelium of the saline group, whereas the positively-stained cells were significantly reduced in the PTX-NaHCO_3_-PLGA NP-treated group (Figure 5C,D). This finding indicated that PTX-NaHCO_3_-PLGA NPs exert a good anti-stenosis effect.

PTX-NaHCO_3_-PLGA NPs can produce CO_2_ in acidic environments, accelerate the release of PTX, and locally release PTX at the injured site, which can concentrate PTX at the injured site, inhibit the migration and proliferation of VSMCs, improve treatment efficiency, and reduce systemic toxicity.

## 4. Conclusions

We successfully constructed a pH-responsive nanomaterial delivery platform using the polymer biomaterial PLGA. In an acidic environment, NaHCO_3_ was able to produce CO_2_ to induce the collapse of PTX-NaHCO_3_-PLGA NPs, thereby inducing the release of PTX, which eventually inhibited the proliferation of VSMCs. In the rat model of balloon-injured vascular restenosis, PTX-NaHCO_3_-PLGA NPs showed better anti-stenosis efficacy and safety than other non-targeted drugs. Therefore, the pH-responsive nanoparticles are potential therapeutic agents for vascular restenosis.

## Data Availability

All available data are reported in the article.
